# Commensal Rodents: Still a Current Threat

**DOI:** 10.3390/pathogens11121483

**Published:** 2022-12-06

**Authors:** Valentina Virginia Ebani

**Affiliations:** 1 Department of Veterinary Sciences, University of Pisa, Viale delle Piagge 2, 56124 Pisa, Italy; valentina.virginia.ebani@unipi.it; 2 Centre for Climate Change Impact, University of Pisa, Via del Borghetto 80, 56124 Pisa, Italy

Commensal rodents live in human habitats where they can find essential elements, including food, water, shelter, and space. The house mouse (*Mus musculus*), Norway rat (*Rattus norvegicus*), and roof rat (*Rattus rattus*) are the main rodent species categorized as commensal. All are responsible for several problems. Rodents often gnaw through almost any material, including lead and cement, causing expensive structural damages; they also are considered as causes of fires when they gnaw on electrical wires. Rodents consume food, partially eating and discarding it, and they spoil agricultural crops. 

Farms are environments that are often heavily infested by commensal rodents that cause damages to structures and animals, with consequent relevant economic losses. In farms, they find food and protection against adverse climatic conditions during very cold or hot times of the year. In particular, poultry farms are frequently infested by rodents that damage eggs, egg trays, gunny bags, poultry feed, and poultry house structures [1]. The large presence of rodents is the cause of decreased poultry performance as well [2]. In fact, they not only cause high mortality rates mainly by killing baby chicks but also induce production losses by breaking and/or eating eggs and frightening birds by their movements or by the noises they create [3]. 

Alongside all these problems that have a relevant economic impact, there is the sanitary aspect involving human and animal health. Commensal rodents contaminate environments and foodstuffs with different pathogenic virus, bacteria, and parasites via their urine, feces, and hairs. In addition, these pests often carry fleas, ticks, and other hematophagous arthropods, which potentially spread diseases, such as Lyme disease by *Borrelia burgdorferi*, murine typhus by *Rickettsia typhi*, and the bubonic plague by *Yersinia pestis*. Furthermore, recently, some spotted fever group *Rickettsia* species have been detected in rodents, suggesting them as natural reservoirs for these bacteria [4].

Bacterial infections in people and animals caused by rodents have been reported globally. The transmission of zoonotic diseases from rodents is possible by direct contact, bites, indirect contact with contaminated objects, oral ingestion, or the inhalation of aerosolized bedding, feces, and urine. Rat bite fever, tularemia, leptospirosis, salmonellosis, yersiniosis, campylobacteriosis, and pathogenic E. coli infections are well-known bacterial zoonoses responsible for infections, and they sometimes have lethal outcome that are more or less frequent in relation to the geographic area and hygienic conditions.

*Francisella tularensis*, the etiologic agent of Tularemia, is largely distributed to the Northern hemisphere (Europe, North America, and Asia) and is not normally found in the tropics or the southern hemisphere, although it has been isolated in Australia [5]. Rodents and lagomorphs, excreting francisellae in secretions and excreta, are sources of infections for humans, who become infected when ingesting contaminated drinking water, inhaling contaminated aerosols or agricultural and landscaping dust, having skin contact with infected animals, and also receiving bites of ticks and deer flies carrying the bacteria [6].

Rat bite fever is caused by *Streptobacillus moniliformis* and *Spirillum minus* in North America and Asia, respectively, where people typically become infected after contact with rodents or via the consumption of food or water contaminated with the urine and droppings of rodents carrying the bacteria [7].

Leptospirosis is a global zoonoses traditionally associated with rodents; they are most frequently infected by *Leptospira interrogans* serovar Icterohaemorrhagiae, but other serovars may be excreted by these animals. Rodents act as maintenance hosts for leptospirae and play a pivotal role in the epidemiology of leptospirosis contaminating environments with their urine. Increased cases of leptospirosis have been related to more frequent episodes of heavy rain that favor animal urine in the soil or on other surfaces to run into floodwater, contaminating it [8]. 

Commensal rodents are also sources of bacterial enteropathogens, and they are often antimicrobial resistant, such as *Salmonella* spp. and other Enterobacteriaceae and *Campylobacter* spp. [9]. Infected rodents excrete these pathogens in their feces, contaminating different environments, including farms, kennels, and gardens. Furthermore, they may serve as microbiological hazard transmitters for predators such as birds of prey, wild canids, and cats. In all cases, commensal rodents become the direct or indirect sources of infections by enteropathogens for humans as well.

Recently, Platt-Samoraj and coworkers [10] demonstrated that rodents can be vectors of different *Yersinia* species responsible for the contamination of farm facilities. *Yersinia enterocolitica* is the most widespread species, but *Y. kristensenii* and *Y. pseudotuberculosis* have been found in the feces of rodents as well.

In conclusion, nowadays, commensal rodents are a severe threat globally not only in geographic areas with poverty and poor hygiene conditions but also in countries with good economic statuses. All people know the material damages caused by rodents, but only a few persons are adequately informed about the diseases and their severity, which are transmissible by these pests. Control measures against commensal rodents by maintaining good hygiene conditions and using traps and rodenticides are required in all environments with features of food availability and temperatures that attract pests.
